# Detecting and Preventing Sybil Attacks in Wireless Sensor Networks Using Message Authentication and Passing Method

**DOI:** 10.1155/2015/841267

**Published:** 2015-07-05

**Authors:** Udaya Suriya Raj Kumar Dhamodharan, Rajamani Vayanaperumal

**Affiliations:** ^1^Department of Computer Science and Engineering, Sathyabama University, Chennai, Tamil Nadu 600 119, India; ^2^Department of Electronic and Communication Engineering, Veltech Multitech Dr. Rangarajan Dr. Sakunthala Engineering College, Avadi, Chennai, Tamil Nadu 600 062, India

## Abstract

Wireless sensor networks are highly indispensable for securing network protection. Highly critical attacks of various kinds have been documented in wireless sensor network till now by many researchers. The Sybil attack is a massive destructive attack against the sensor network where numerous genuine identities with forged identities are used for getting an illegal entry into a network. Discerning the Sybil attack, sinkhole, and wormhole attack while multicasting is a tremendous job in wireless sensor network. Basically a Sybil attack means a node which pretends its identity to other nodes. Communication to an illegal node results in data loss and becomes dangerous in the network. The existing method Random Password Comparison has only a scheme which just verifies the node identities by analyzing the neighbors. A survey was done on a Sybil attack with the objective of resolving this problem. The survey has proposed a combined CAM-PVM (compare and match-position verification method) with MAP (message authentication and passing) for detecting, eliminating, and eventually preventing the entry of Sybil nodes in the network. We propose a scheme of assuring security for wireless sensor network, to deal with attacks of these kinds in unicasting and multicasting.

## 1. Introduction

A wireless sensor network consists of applications such as environmental monitoring, target tracking, health monitoring, and other various maintenance options. Implementation and topology creation have become significant activities in modern research work [1]. The usage of wireless sensor network in a variety of applications is highly important with the emphasis on ensuring security. Still, Prevention and detection of malicious attacks of all levels may be high or low in wireless sensor network [2]. A variety of attacks on the network like wormholes, sinkhole, Sybil, sleep, and selective forward attacks in the network are being observed. Many researchers have identified their own infrastructures which have portable devices, used in various trade services in decentralized and scalable methods. Some of the devices are capable of synchronization without the use of the internet for multiuser applications. They are used for finding the exact location in the algorithms that enhance the accuracy. The Sybil attacker misleads other nodes by showing wrong ID or duplicate ID of the users who are aware of the nodes in the wireless sensor network.

In the latest network environment, alien nodes can appear in disguise in various identities and act as original nodes. Basically, there is no common master node in social and defense network for monitoring communication between network nodes intense [3]. The analysis of peer-to-peer network shows that these networks show the existence of these network logical functionalities or the virtual networks coventry exist, that is, the networks built on the top of other networks as in the internet. The network node addresses are based on the logical ID for structuring and forming networks [4].

The nodes in wireless sensor network are not in a fixed infrastructure, whether single-hop, multihop communication, base station, gateways, and access points [5]. Basically, wireless sensor networks have a smaller infrastructure which could be noninfrastructure networks. The term ad hoc implies the establishment for a special purpose and for applications such as tracking, function approximation and edge detection, monitoring environment, and security domain in the homeland. The application of wireless sensor network, resembling a military force, monitors absence of restriction on the infrastructure as well as in the intermediate hop nodes [6].

This paper deals with one of the hazardous security threats known as Sybil attack and proposes an algorithm known as message authentication and passing method to hinder a Sybil attack in a wireless sensor network. The rest of the paper is presented as follows: Section 2 defines the materials and methods of the Sybil attack; Section 3 defines the results and discussions.  Section 4 provides conclusions and indications of future work.

## 2. Materials and Methods

Sybil attack is a matter of critical importance and consternation in network security leading to many fake identities that can cause disruption in the network [7]. Sybil attack occurs mostly during broadcasting and it functions without individual verification and identity comparison of communication entities [8]. The attacker node can acquire many identities. That entity in the system can endeavor to influence the Sybil attacker due to the awareness of only others in each entity via messages in the communication channel [9]. The attacker nodes are launched inside and outside the route as well as wireless sensor networks. The monitoring node specially identifies the attacker node on a unicast as well as in a multicast scenario. Here, [10] author proposes an authentication framework which can ensure hindrance to or mitigation of security attacks on wireless sensor network.

### 2.1. Security Attacks on WSN

Various types of malicious activities are patent in wireless sensor network. Some of these are created in terms of nodes while others are created in a network, data link, and application layers. Some are created in the physical state [11].

The attacks are currently classified as active and passive. The former is created by deployment of illegal information in the network that can affect it. Sybil, sinkhole, and eavesdropper are some of the active attacks. Passive attacks are those which are meant to affect the network resources such as lifetime and network size.

### 2.2. Sybil Attack

**Figure 1:**
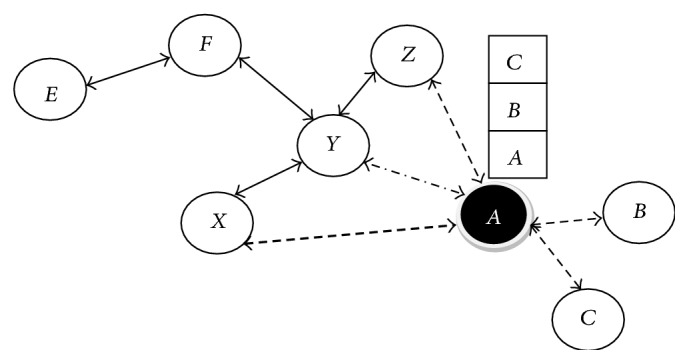
Sybil attacks with multiple ID.

A node or a device takes many identities that may not necessarily be lawful. It does not impersonate any node, but fast it only assumes the identity of another among several nodes, causing redundancies in the routing protocol. Sybil attacks degrade data integrity, security, and resource utilization. It can also perform storage, routing mechanisms, air resource allocation, and misbehavior detection. In a sensor network hundreds of sensor nodes form the communication network. The wireless communication between these sensor nodes passes through a central station. These nodes communicate with a specified of nodes of a specified number [12]. There are many encryption techniques available to prevent external attack on the nodes, but nodes in the communication network can also mount an attack. One of these insider attacks is called a Sybil attack [13–15] in which the node that spoofs the other node is called Sybil node *S* and the other one is a normal node *N*. In a proper communication system only *N* nodes should communicate with one another. But here, *S* node comes in another form of its own as an internal known node and launches an attack on the network. The Sybil node tries to communicate with neighboring nodes by using the identity of the normal node and in the process a single node gives many identities in the area to other nodes in the network which is illegal. A Sybil node can be formed as a new identity or as a pilfering legal identity. It is, therefore, considered an additional entity of a misbehaving node. This causes confusion in the network and it gets collapsed. A faulty node which enters into the network with different IDs is shown in Figure 1.

As a result Sybil attacks are classified into two forms on the basis of the manner of attack on the network. They are as follows.

*(i) Direct Attack and Indirect Attack*. In a direct attack, the real nodes communicate directly with Sybil nodes, whereas, in an indirect attack, the communication is done through a malicious node.

*(ii) Fabricated Attack and Stolen Identity Attack*. Legal identities of nodes are used to create new illegal nodes. That is to say, a sensor node which has an ID of 16-bit integers creates the same ID of 16 bits, which are fabricated nodes. The IDs stolen by the Sybil node are destroyed by checking the identity replication [16].

### 2.3. Existing Methodology

Random Password Generation (RPC) algorithm focuses on the various traffic levels and security during data transmission in WSN. RPC algorithm generates the routing table which holds information about deployed nodes. The intermediate nodes in the route are identified between source and destination. The intermediate node's information is compares with RPC database during communication among nodes, based on the comparison results it decides whether Sybil or normal node. RPC also generates the route by adding the genuine node in its path from source to destination node using several subprocedures [17].

### 2.4. Proposed Approach

The main objective of this paper is to design and develop an algorithm for detecting and preventing Sybil attacks in wireless sensor network. It is referred to message authentication and passing algorithm. Creation of Sybil activity through use of the other personal identities is well known. Most of the existing research deals with the detection of the Sybil attack through verification of identities.

#### 2.4.1. Network Model

In this paper, *N* numbers of nodes are deployed in the network randomly under the control of and an administrator. These are well configured, energy efficient, and promising nodes in the network. During node creation, each node will receive a *HELLO* message from the *BS* with a timestamp message indicating the node creation time (birth time) in the network. The entire node responds to the BS with a RES message with ID, timestamp, and location. Then this information is stored in a *iNODEINFO*_*table* under the control of the administrator of the network. The entire network model is presented as *G* = {(*N*1, *N*2,…, *Ni*,…, *Nm*), *BS*, Admin} where *m* is the number of nodes in the network. Each node is deployed in the network as Location(*Ni*) = (rand⁡(*x*), rand⁡(*y*)), where *X*, *Y* is any location within the network area. The* BS* sends a HELLO packet to all the newly created nodes in the network which can be written as

(1) BSMsg,τ∑i=1mNi,where  N1,N2,…,Ni,…,Nm  are  Nodes.

And, each node in the network is sending a* RES* packet to the* BS* which can be written as *Ni RES BS*, where the HELLO and* RES* packet consist of node ID and the timestamp. HELLO = *τ*(*Ni*) and *RES* = (ID(*Ni*), *τ*(*Ni*)), where *Ni* denotes the *i*th node, *τ*(*Ni*) denotes timestamp of the *i*th node, and ID(*Ni*) denotes identity of the *i*th node. The parameters such as ID and *τ* are used to verify that the node is a Sybil or not. CAM-PVM algorithm for Sybil detection.

In network *G*, a node *S* needs to transmit a data to a node *D*. So, it is necessary to discover a route from *S* to *D* through an *N*-hop intermediate node. The number of intermediate nodes depends on the network size. The routing mechanism used in this paper follows AODV protocol. During this process, current information about the intermediate nodes (ID, timestamp) is tentatively stored in a routing table named as *iROUTING*_*table*.

The duration between the route discovery and data transmission in the discovered route is very small. While data transmission, the *iROUTING*_*table* data entries are compared with the entries available in the *iNODEINFO*_*table* shown in Table 1, where it helps to identify the duplicate nodes with id, timestamp, and the location. For example, *G* = {*N*1, *N*2,…, *Ni*,…, *Nm*}*N*3 is considered source node, *N*9 the destination node, and the intermediate nodes are *N*5, *N*7, and *N*8. The route discovered from *N*3 to *N*9 is *N*3 → *N*5 → *N*7 → *N*8 → *N*9. The *iROUTING*_*table* of the discovered route is shown in Table 2, which comprises the original node ID, timestamp [*τ*], location, and the current timestamp during the time of route discovery.

Now during the data transmission, the discovered route is verified by comparing current intermediate node information with the *iROUTING*_*table* by updating the node entries. From Table 3, it is clear that the *N*7 information is replicated; it is found that the information of the replica node does not match the original *N*7 information in *iNODEINFO*_*table*.

Sybil activity is identified with application of the CAM-PVM algorithm and is detected in the network. To provide prevention for Sybil activity, another MAP algorithm is applied along with CAM-PVM for prevention of Sybil activity. MAP comprises unicast as well as multicast based communication in the network. The algorithm for CAM-PVM and MAP is given below.

*Compare and Match-Position Verification Method (CAM-PVM)*

1. *Let* *G* = {*N*1, *N*2, *N*3,…, *Nm*}
2. *Let* *BS*, *Admin* *be* *the* *well* *configured* *nodes*
3. *for* *i* = 1  *to* *m* // Nodes are placed
4. randomly
5. *Ni* ← Location(rand⁡(*X*), rand⁡(*Y*))
6. ID(*Ni* ← *i*);
7. *End* *Loop*
8. * Let Li be the set of link between pair of nodes in the network*
9. *Ni* → *BS*(Msg, *τ*)// For every nodes *Ni*
10. *U* ^*∗*^ → *minimum*  distance(*Ni*, *Ni* + 1)
11. *Ni* *RES* *BS*; *RES* = ID, *τ*, *X*, *Y*;
12. *RES* → *iNODEINFO*_*table*
13. *End* *Loop*
14. *Su*{U^*∗*^} → *S*
15. *S* → *RT*(*S*)
16. *Ni* + 1 → *Ni*(*τ*)
17. *τc* = (ID, *X*, *Y*, *τ*)
18. *U* ^*∗*^ → *D*
19. *for* *i* = *S* *to* *D* //Route Discovery
20. *RT*(*S*)  *Ni*;
21. Li → (*Ni*, *Ni* + 1);
22. *RES* → *iROUTING*_*table*
23. *End* *Loop*
24. *for* *i* = *S* *to* *D*// Data transmission
25. *if*(*current* *Ni*.*info* = =
26. iROTINGtable==iNODEINFOtable)then
27. ifcurrent  Ni.info==iROUTINGtable==  iNODEINFOtablethen
28. *Ni* *data* *Ni* + 1
29. *else*
30. *Ni* + 1  *is* *blocked* *as* *Sybil* *Node*
31. *End* *Loop*
32. *iROUTINGtable* entries clear
33. End

The CAM-PVM algorithm is used during the discovery and data transmission in the network, where the node's information is checked from the* BS iNODEINFO*_*table*. After verification of CAM-PVM algorithm, the algorithm collects the ID, timestamp, and current location information of the nodes and compares with initial information when they are registered. The results of the CAM-PVM algorithm can provide only the trusted nodes in the route to ensure secured data transmission. Otherwise the particular nodes are treated as unknown nodes such as Sybil and data transmission in the current is stopped and alternate path is selected.

Application of CAM-PVM is a time consuming process and also cost effective. So, that prevention device is suggested in this paper to eliminate Sybil activity. Each node should communicate by passing the authentication message. In case the source node suspects the destination dynamically, we can make use of the CAM-PVM with MAP algorithm for comparing and for message authentication to check whether the current node is Sybil or not. Where in the network *G*, a node *Ni* passes the data to node *Nj* the node *Ni* sends a request message to node *Nj* with its key, as msg(*Ni*), which is generated by the* BS* while registering in the network *G*. Node *Nj* (destination node) submits its key message with msg(*Nj*), and later, both keys are verified by the base station and an ok signal produced for sharing the data and any other information. Data transmission occurs between *Ni*, *Nj* once they get the signal from the base station. The pseudocode uses for the message authentication and passing method are given below in detail.

*Message Authentication and Passing (MAP)*

1. *G* = {*N*0, *N*1, *N*2,…, *Nn*}
2. *for* *I* = 1  *to* *n*
3. *r*table = addrec(*Ni*(id), *Ni*(*x*), *Ni*(*y*), *Ni*(msg))
4. // node id, *x*, *y* values of *i*th node
5. *End*
6. *for* *I* = 1  *to* *n*
7. *for* *J* = 1  *to* *n*
8. *Ni* → *Send*(*request*) → *Nj*
9. *Nj* → *Send*(*acceptance*) → *Ni*
10. *If*(msg(*Ni*), msg(*Nj*))*exists*(*rtable*)) then
11. *Ni* → *Send*(*dataP* → *Nj*)
12. *Else*
13. *Choose* *the* *next* *neighbor*
14. *End* *If*
15. *End* *for* *J*
16. *End* *for* *I*
17. End

## 3. Results and Discussion

**Figure 2:**
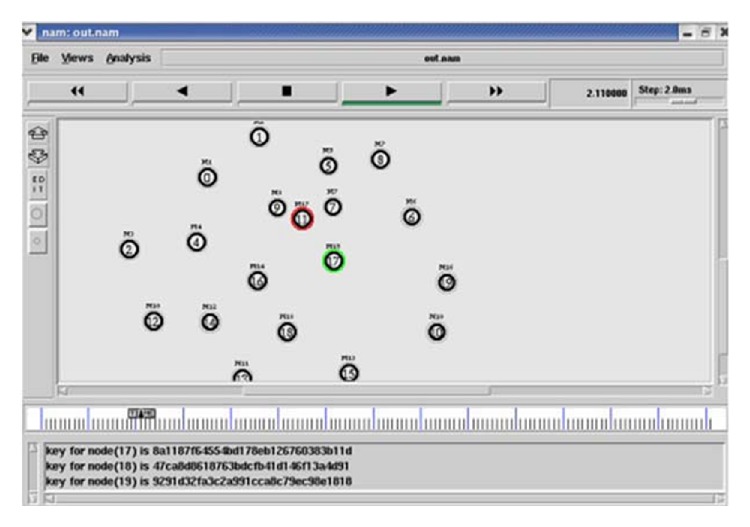
Simulation of identifying Sybil node.

The entire system model is simulated using NS2 software with 25 nodes with a network size of 1200 × 1200. Each sensor node behaves under AODV (Ad Hoc on Demand Vector) protocol. All nodes are constructed under a single* BS*. In this network model, node 17 is ready to receive the data from node 0. When node 0 sends a REQ message to node 17, that node sends a* RES* message to node 0 back. This can be sensed by node 11 which sends a* RES* message with the label of node 17. This can be traced while node 11 is unable to submit its authenticated key value which belongs to node 17. So it is detected as a Sybil node and rejects node 11 from the network. In this paper, the efficiency of the network is calculated by comparing the throughput before and after inclusion of message authentication and passing algorithm in the network functionality. This is described in detail in Figure 2.

**Figure 3:**
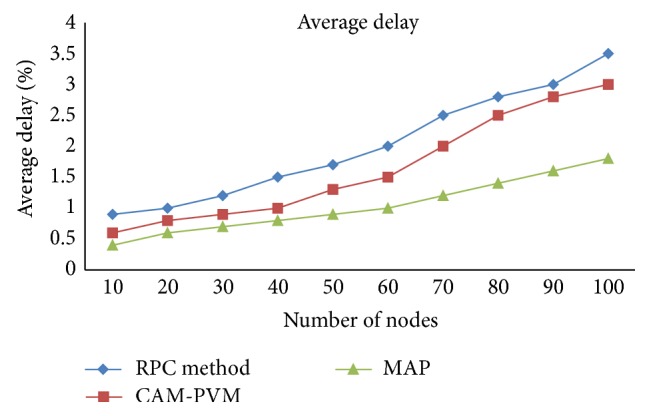
Comparison of average delay of data packet transfer between existing method RPC with CAM-PVM and MAP.

As the proposed approach is meant to ensure detection and prevention of the Sybil node, the performance can be analyzed by calculating the average delay of data packet transfer, throughput, malicious node, and other necessary factors for judging the quality of service of a routing protocol. In this paper, Figure 3 shows the average delay of the data packet transfer of the network before deployment of the message authentication and passing algorithm where data packets of different sizes are transmitted at different intervals of time. The behavior of Sybil nodes resembles dispatch of data at any time from any location and that would disturb the original nodes in the network. The figure shows the average delay is very less after applying the MAP algorithm.

**Figure 4:**
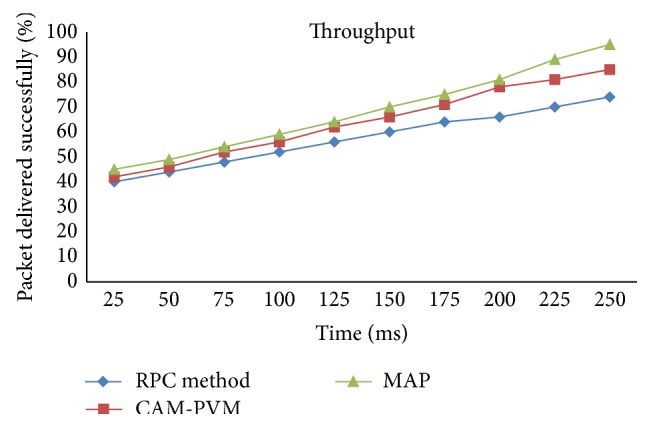
Comparison of throughput between existing RPC method with CAM-PVM and MAP.

In order to check the performance evaluation, a large number of nodes are deployed in the network and the detection rates of the Sybil nodes. A number of iterations can be made with different numbers of nodes in the network as shown by the performance. With the increase in the number of nodes, there is also an increase in the number of misbehaving nodes, which obviously affects the data and results in data loss. The throughput of the proposed approach before and after deployment with the routing protocol is calculated. In existing Random Password Comparison method, the throughput will be 74% whereas in case of CAM-PVM it will be 85% and in MAP it will be 95%. It shows the efficiency of the proposed approach. The comparison of throughput between existing method with CAM-PVM and MAP is shown in Figure 4.

Once a detection procedure is deployed during transmission, it detects the Sybil node and avoids transmission through that attacker node. Data loss can be thwarted through detection. In this scenario the number of times for consumption should be considered for the improvement of the quality of service. The detection rate of the Sybil attack is more accurate, but considering waste of time in such a kind of situation, prevention as a factor that directly eliminates the Sybil nodes is the deciding factor in the place of detection. Subsequent elimination of the procedure message authentication and passing method for prevention of the Sybil attack is applicable, but the detection rate is smaller compared to CAM-PVM and other existing methods. There is a clear indication of this fact. The same simulation is repeated for *n* number of nodes with *n* number of times in network simulation software.  Table 4 shows the comparison of CAM-PVM with message authentication and passing method.

**Figure 5:**
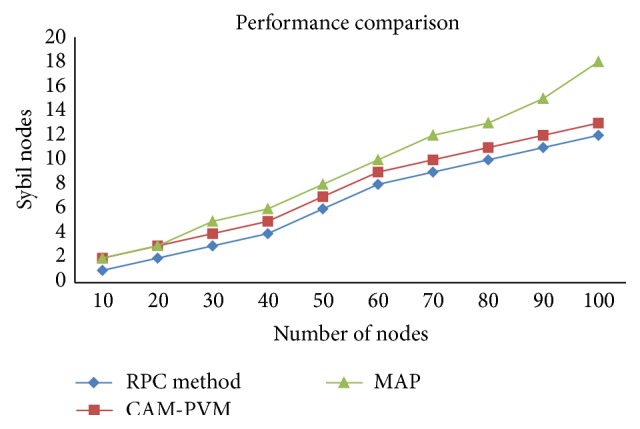
Number of Sybil node detection with existing RPC method with proposed between CAM-PVM and MAP.

Simulation is also carried out in multiple rounds where the number of nodes deployed that are different for each round and there is a difference in the number of Sybil nodes according to the normal nodes. Here we had conducted a simulation of our proposed algorithm by assigning 2 Sybil nodes for each 10 nodes and forward our simulation process up to 100 nodes starting from 10 nodes.  Table 4 shows our simulation result for Sybil node detection from which we can clearly say that our MAP algorithm produces 30% more detection accuracy compared with the CAM-PVM algorithm and existing RPC methods. In case of least Sybil nodes both algorithms produce same results but when the number of malicious nodes increased the performance of CAMPVM gets decrease while our algorithm maintains its consistency. The performance is comparatively good in message authentication and passing method. The optimized and compared output of the MAP with CAM-PVM algorithm and existing RPC method is given in the graphical representation as shown in Figure 5.

**Figure 6:**
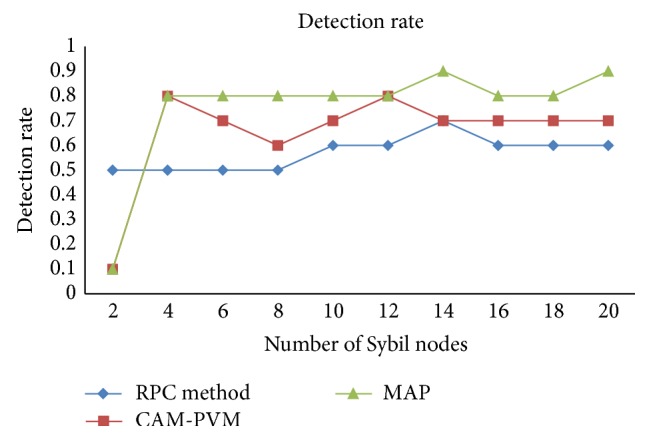
Comparison of detection rate with existing RPC method with proposed CAM-PVM and MAP.

Figure 6 shows the comparison of detection rate with existing RPC methods with proposed CAM-PVM and MAP. In existing RPC method, the detection rate was only 60% whereas in proposed CAM-PVM the detection rate was 75% and in MAP the detection rate was 90%. It shows the efficiency of the proposed approach. The performance comparisons show three categories in which the performance values are compared using the existing methodology. The three factors are data packet transfer; throughput and detection of malicious node are computed and compared. In the first methodology, average delay of data packet transfer between existing RPC method with proposed method. The average delay is calculated as successful data packet transmission. In the second methodology throughput is calculated between the source nodes to destination node. By comparison with the existing system throughput will be 74% whereas in CAM-PVM is taking 85% and message authentication and passing method 95% of the total time. In the third methodology, the detection of malicious node comparison between the existing system is 60% whereas in CAM-PVM detects 65% of malicious node whereas the message authentication and passing method detects 90% of the malicious node. Hence message authentication and passing method is considered a better method than the CAM-PVM even under this criterion.

## 4. Conclusions

In this paper the message authentication and passing method is applied for checking the trustworthiness or otherwise for a Sybil node. The action of a node as a Sybil node with duplicate ID and information can happen only when the node has complete information about other nodes. Verification of the node needs the application of CAM-PVM. Instead of wasting time for CAM-PVM to check each and every node, the message authentication and passing procedure is applied for authentication prior to communication. If a node does not have any authorization by the network or by the base station, it cannot communicate with any other node in the network. The message authentication and passing method is so effective and is known for more time consuming than any other method.

Message authentication and passing method requires modification and reduction in time consumption and for cost effectiveness. The size of the network is not a constraint. The throughput of the network should be higher than the other security algorithm which is applied earlier in the network security.

## Figures and Tables

**Table 1:** *iNODEINFO_table*.

Node ID	*N*1	*N*2	*N*3	*N*4	*N*5	*N*6	*N*7	*N*8	*N*9	*N*10
*τ*	11:00	11:02	11:30	11:37	11:41	11:43	11:56	11:59	12:04	12:06
*X*	34	67	87	123	154	23	18	54	62	59
*Y*	11	33	54	67	89	123	131	145	165	154

**Table 2:** *iROUTING*_*table*.

Node ID	*N*3	*N*5	*N*7	*N*8	*N*9
*τ*	11:30	11:41	11:56	11:59	12:04
*X*	87	154	18	54	62
*Y*	54	89	131	145	165
*τ*	1:01	1:04	1:05	1:06	1:06

**Table 3:** Node *N*2 is the Sybil, acting as *N*7.

Node ID	*N*3	*N*5	*N*7 [*N*2]	*N*7	*N*8	*N*9
*τ*	11:30	11:41	11:02	11:56	11:59	12:04
*X*	87	154	67	18	54	62
*Y*	54	89	33	131	145	165
*τ*	1:01	1:04	1:05	1:05	1:06	1:06

**Table 4:** Comparison of Sybil node with existing RPC method with CAM-PVM and MAP.

Number of nodes	10	20	30	40	50	60	70	80	90	100
Sybil nodes	2	4	6	8	10	12	14	16	18	20
RPC	1	2	3	4	6	8	9	10	11	12
CAM-PVM	2	3	4	5	7	9	10	11	12	13
MAP	2	3	5	6	8	10	12	13	15	18

## References

[1] Rathod V., Mehta M. (2011). Security in wireless sensor network: a survey. *Ganpat University Journal of Engineering & Technology*.

[2] Modirkhazeni A., Ithnin N., Abbasi M. (2012). Secure hierarchical routing protocols in wireless sensor network; security survey analysis. *International Journal of Computer Communications and Networks*.

[3] Niu W., Lei J., Tong E. (2014). Context-aware service ranking in wireless sensor networks. *Journal of Network and Systems Management*.

[4] Baig Z. A. (2011). Pattern recognition for detecting distributed node exhaustion attacks in wireless sensor networks. *Computer Communications*.

[5] Anand D. G., Chandrakanth H. G., Giriprasad M. N. (2012). Security threats & issues in wireless sensor networks. *International Journal of Engineering Research and Application*.

[6] Abbas S., Merabti M., Llewellyn-Jones D. Signal strength based Sybil attack detection in wireless Ad Hoc networks.

[7] Sharmila S., Umamaheswari G. (2012). Detection of sybil attack in mobile wireless sensor networks. *International Journal of Engineering Science & Advanced Technology*.

[8] Ssu K.-F., Wang W.-T., Chang W.-C. (2009). Detecting sybil attacks in wireless sensor networks using neighboring information. *Computer Networks*.

[9] Vasudeva A., Sood M. (2012). Sybil attack on lowest id clustering algorithm in the mobile ad hoc network. *International Journal of Network Security & Its Applications*.

[10] Balachandaran N., Sanyal S. (2012). A review of techniques to mitigate sybil attacks. *International Journal of Advanced Networking and Applications*.

[11] Padmavathi G., Shanmugapriya D. (2009). A survey of attacks, security mechanisms and challenges in wireless sensor networks. *International Journal of Computer Science and Information Security*.

[12] Xiao L., Greenstein L. J., Mandayam N. B., Trappe W. (2009). Channel-based detection of sybil attacks in wireless networks. *IEEE Transactions on Information Forensics and Security*.

[13] Tangpong A. (2010). *Managing sybil identities in distributed systems [Ph.D. thesis]*.

[14] Yu H., Gibbons P. B., Kaminsky M., Xiao F. (2010). SybilLimit: a near-optimal social network defense against sybil attacks. *IEEE/ACM Transactions on Networking*.

[15] Jing-Jing G., Jin-Shuang W., Yu-Sen Z., Tao Z. (2011). Formal threat analysis for ad-hoc routing protocol: modelling and checking the sybil attack. *Intelligent Automation & Soft Computing*.

[16] Komar C., Donmez M. Y., Ersoy C. (2012). Detection quality of border surveillance wireless sensor networks in the existence of trespassers' favorite paths. *Computer Communications*.

[17] Amuthavalli R., Bhuvaneswaran R. S. (2013). Detection and prevention of sybil attack in wireless sensor network employing random password comparison method. *Journal of Theoretical and Applied Information Technologygy*.

